# At-risk individuals display altered brain activity following stress

**DOI:** 10.1038/s41386-018-0026-8

**Published:** 2018-02-26

**Authors:** J. M. C. van Leeuwen, M. Vink, G. Fernández, E. J. Hermans, M. Joëls, R. S. Kahn, C. H. Vinkers

**Affiliations:** 1 Department of Psychiatry, University Medical Center Utrecht, Utrecht, The Netherlands; 2 Experimental Psychology, Utrecht University, Utrecht, The Netherlands; 3 Department of Cognitive Neuroscience, Donders Institute for Brain, Cognition, and Behaviour, Nijmegen, The Netherlands; 4 Department of Translational Neuroscience, University Medical Center Utrecht, Utrecht, The Netherlands; 5 University Medical Center Groningen, University of Groningen, Groningen, The Netherlands

## Abstract

Stress is a major risk factor for almost all psychiatric disorders, however, the underlying neurobiological mechanisms remain largely elusive. In healthy individuals, a successful stress response involves an adequate neuronal adaptation to a changing environment. This adaptive response may be dysfunctional in vulnerable individuals, potentially contributing to the development of psychopathology. In the current study, we investigated brain responses to emotional stimuli following stress in healthy controls and at-risk individuals. An fMRI study was conducted in healthy male controls (*N* = 39) and unaffected healthy male siblings of schizophrenia patients (*N* = 39) who are at increased risk for the development of a broad range of psychiatric disorders. Brain responses to pictures from the International Affective Picture System (IAPS) were measured 33 min after exposure to stress induced by the validated trier social stress test (TSST) or a control condition. Stress-induced levels of cortisol, alpha-amylase, and subjective stress were comparable in both groups. Yet, stress differentially affected brain responses of schizophrenia siblings versus controls. Specifically, control subjects, but not schizophrenia siblings, showed reduced brain activity in key nodes of the default mode network (PCC/precuneus and mPFC) and salience network (anterior insula) as well as the STG, MTG, MCC, vlPFC, precentral gyrus, and cerebellar vermis in response to all pictures following stress. These results indicate that even in the absence of a psychiatric disorder, at-risk individuals display abnormal functional activation following stress, which in turn may increase their vulnerability and risk for adverse outcomes.

## Introduction

Stress increases the risk for almost all psychiatric disorders [1]. It is thought that a maladaptive response to stress may impair an individual’s capacity to deal with a demanding environment and that this contributes to the risk for psychopathology [2]. Nevertheless, there are large interindividual differences in outcomes after stressful experiences [3]. Genetic variations influence the neurobiological systems that shape an individual’s response to the environment, and hence determine the degree to which environmental factors such as stress may precipitate the development of psychopathology [4].

Exposure to stress affects behavior and brain functioning in a time-dependent manner [5]. Acute stress rapidly facilitates threat detection and habitual behavior, but inhibits the ability to focus attention and make complex decisions. These types of behavior are accompanied by increased activity within the default mode network (DMN) [6] and salience network (SN) [7]. In the aftermath of stress, the stress hormone cortisol plays a major role in the normalization in emotional reactivity with concomitant decreases in the SN [5, 8,] and DMN [9]. This dynamic shift in brain functioning during and following stress is hypothesized to underlie an adaptive stress response. It has been hypothesized that this adaptive response can become maladaptive in vulnerable individuals and lead to psychopathology [10]. However, studies investigating the effects of stress on the brain of at-risk individuals are relatively scarce.

In this study, we therefore investigated emotion processing half an hour after stress in healthy male individuals and unaffected siblings of schizophrenia patients. Siblings of schizophrenia patients are at risk for a wide range of psychiatric disorders including schizophrenia, depression, and bipolar disorder [11] and show increased sensitivity to daily life stress compared to healthy controls [12]. Even in the absence of stress, individuals at increased risk for schizophrenia show impaired emotion processing and regulation [13]. This may in turn increase their vulnerability for the detrimental effects of stress. We hypothesized that, following stress, healthy controls would exhibit a shift toward decreased activity in the SN and DMN to emotional images, whereas this shift would be impaired in at-risk individuals.

## Methods and materials

### Participants

A total of 40 healthy male siblings of schizophrenia patients (referred to as “siblings” hereafter) and 40 healthy male controls were recruited from the Genetic Risk & Outcome of Psychosis (GROUP) study [14] (4 controls, 6 siblings) and via advertisements (36 controls, 34 siblings). Current psychiatric disorders were excluded in all individuals using a semi-structured interview by a trained researcher (The Mini-International Neuropsychiatric Interview (MINI)) [15]. Furthermore, healthy controls did not have first-degree relatives with a psychiatric disorder. Participants suffering from a neuroendocrine disorder or claustrophobia were excluded. None of the participants had been working night shifts in the week preceding participation or were using any corticosteroids or antipsychotics, which are known to influence the cortisol response [16]. Participants were randomly assigned to the stress or no-stress condition of the trier social stress test (see below for detailed description) using block randomization (block size = 4). Two subjects were excluded due to technical problems with the MRI scanner. This resulted in four experimental groups: control-no-stress (*n* = 19), control-stress (*n* = 20), sibling-no-stress (*n* = 20), and sibling-stress (*n* = 19). The number of subjects did not vary across analyses.

Participants were instructed to refrain from drugs (2 weeks prior to participation), alcohol (24 h prior to participation), heavy exercise (2 h prior to participation), and caffeine (4 h prior to participation). Current use of psychoactive substances (amphetamines, cocaine, opiates, methadone, benzodiazepines, and cannabinoids) was determined with a urine multidrug screening device (multiline) and self-report questionnaire. Two subjects (1 control and 1 sibling) scored positive for cannabis. Exclusion of these participants did not influence any of the results. Participants that smoked tobacco daily were defined as smoker. Prior to the experiment, all participants gave written informed consent. All procedures were checked and approved by the Utrecht Medical Center Utrecht ethical review board and performed according to the guidelines for Good Clinical Practice and the declaration of Helsinki.

### General procedures and stress induction

All participants were told that they were taking part in a study on the effects of “cognitive load” on the brain and that not all the information regarding the study purpose could be provided. The trier social stress test (TSST) was carried out as previously published [17]. In short, participants received instructions 5 min prior to the stress or control condition, which was carried out outside the scanner in a separate room. The stress condition consisted of a 5 min job interview, followed by a 3 min mental arithmetic task in front of a committee (one woman and one man). The committee was instructed to act the same for all subjects. The validated control condition consisted of a free speech (5 min) followed by a simple arithmetic task (3 min) [18]. The experimenter was in the same room but did not evaluate the participant, nor was there a committee present. The TSST was carried out between 4:30 and 8:30 p.m. to minimize variation in diurnal cortisol secretion. The picture task was carried out on average 33 min after TSST onset.

### Picture task

The picture task consisted of the presentation of pictures from the International Affective Picture System (IAPS) as previously published [19]. Pictures belonging to three categories were shown: neutral, negative, and positive, according to validated ratings of the IAPS. Pictures were matched on arousal rating and shown in pseudo-random order during four blocks (eight pictures of each condition) interleaved with four rest blocks (attending to a fixation cross). Each condition consisted of 32 pictures. Each picture was presented for 2 s. After 2 s, participants had to rate the picture (neutral, negative, or positive) by pressing a button, after which a fixation cross appeared for the remaining trial duration (maximum 2 s). A full description of the task can be found in ref. [13].

### Salivary cortisol and alpha-amylase assessment

In total, seven saliva samples were obtained throughout the experiment using salivettes (Sarstedt, Nümbrecht, Germany) for the quantification of cortisol and alpha-amylase. Salivary cortisol concentrations are highly correlated with free cortisol (the proportion biologically active cortisol) concentrations in the blood [20]. Alpha-amylase, an enzyme secreted by the salivary gland, is a marker for (nor)adrenergic activity and is only found in saliva, not in blood [21]. Samples were obtained −10, +5, +20, +30, +65, +90, and +120 min relative to TSST onset. Samples were directly stored at −20 °C and cortisol and alpha-amylase levels were analyzed as previously described [22]. The cortisol area under the curve with respect to the increase (AUCi) was quantified as previously described in ref. [23]. Three out of 546 samples were missing (all non-peak values) and were calculated by the mean group decline. Exclusion of participants with missing data did not affect any of the results. The alpha-amylase percentage increase was based on the change from the first (before TSST) to the second (during TSST) sample.

### Questionnaires

To assess exposure to stress prior to the study, participants completed data on validated childhood trauma (CTQ, Dutch version [24]) and major life events (LSC-R [25] questionnaires). During the experiment, subjective stress was assessed using a 100 mm visual analog scale (VAS), which was completed before, during and after the stress or control test (−10, +5, and +20 min after onset).

### Functional MRI

All imaging was performed on a Philips 3.0-T whole-body MRI scanner (Philips Medical Systems). First, a whole-brain three-dimensional T1-weighted structural image was acquired with the following scan parameters: voxel size 1 mm isotropic; repetition time (TR) = 10 ms; echo time (TE) = 4.6 ms; 200 slices; flip angle = 8°. Functional images were obtained using a two-dimensional echo planar imaging-sensitivity encoding (EPI-SENSE) sequence with the following parameters: voxel size 3 mm isotropic; TR = 2000 ms; TE = 35 ms; 30 slices; gap = 0.43 mm; flip angle = 70°. Two hundred fifty-six dynamic scans were acquired during the task (acquisition time: 8 min 30 s).

### Image preprocessing

First, data were realigned, and corrected for differences in acquisition time between slices, co-registered, spatially normalized into standard stereotactic space (Montreal Neurological Institute, MNI, 152 space), and spatially smoothed using a 6-mm FWHM Gaussian kernel to minimize noise and residual differences in individual neuroanatomy.

### Statistical analyses

#### Cortisol and alpha-amylase

For changes in cortisol level over time, the effects of stress (stress/no-stress) and group (control/sibling) were analyzed using a repeated measures analysis of variance (ANOVA). For the AUCi and alpha-amylase percentage change, we used a two-way ANOVA using SPSS 23.0 (Statistical Package for the Social Sciences, Chicago, IL).

#### Behavior

We performed two repeated measures ANOVAs to test for effects of valence (neutral, negative, and positive), stress, and group on rating accuracy reaction time of the trials as well as head movement using SPSS 23.0.

#### fMRI

Imaging data were analyzed using SPM8 (http://www.fil.ion.ucl.ac.uk/spm). The effects of picture valence (neutral/negative/positive) on brain activity were estimated during individual first-level analyses. A detailed description of the first-level analysis can be found in ref. [19]. In short, we only included trials in which the participant’s response corresponded to the IAPS rating to improve detection of emotion-related brain activation [26] (see Table S1 for percentage accurate trials for each valence and group). Subsequently, the design matrix consisted of three regressors modeling the onsets and duration (2 s) of the neutral, negative, and positive trials. These factors were convolved with a canonical hemodynamic response function. The realignment parameters (three translations and three rotations) obtained from slice-time correction were added as factors to correct for head movement. A high-pass filter with a cutoff period of 128 s was applied to correct for signal drift. We chose to study the effects of stress on negative, neutral, and positive pictures versus rest, rather than subtracting neutral images from negative and positive pictures as described in ref. [19], because a previous study has found valence-independent effects of cortisol on brain responses. However, since they only used negative and positive stimuli, it is unknown whether the effects are restricted to emotional stimuli, or that the effects are similar across emotional and neutral images. Analyzing all three valences separately may provide an answer to this question. Moreover, subjects with a (genetic vulnerability for) psychiatric illness process neutral faces different than healthy controls [27, 28,]. Therefore, the use of neutral stimuli (some of which were neutral faces) as a baseline control condition may affect the interpretation of results and conceal the true difference in emotion-related brain responses.

On the group level, we investigated whether siblings showed different activation patterns after stress than controls. First, we assessed the interaction between group, stress and picture valence using a 2 × 2 × 3 full factorial ANOVA with group (control/sibling) and stress (stress/no-stress) as between-subject factors and picture valence (neutral/negative/positive) as within-subject factor. Subsequently, we investigated the group × stress interaction independent of stimulus type using a 2 × 2 full factorial ANOVA with group (control/sibling) and stress (stress/no-stress) as between-subject factors. Group maps were tested for significance and corrected for multiple comparisons using cluster-level inference (cluster-defining threshold *p* < 0.001, cluster probability of *p* < 0.05 family wise error (FWE) corrected). The anatomical location of peaks was determined on the basis of the neuromorphometrics atlas in SPM (Neuromorphometrics, Inc. http://neuromorphometrics.com/) and previously published coordinates for ventrolateral and medial prefrontal cortices [19, 29, 30,]. If the group×stress interaction resulted in significant clusters, they were subsequently used as data-driven regions of interest (ROIs) for subsequent statistical analyses using Marsbar [31]. The mean regression coefficient (for the factors neutral, negative, and positive in the contrast task versus rest) over all voxels within each ROI was extracted for each subject and analyzed using a 2 × 2 ANOVA with group (control/sibling) and stress (stress/no-stress) as between-subject factors. Post hoc group comparisons were Bonferroni corrected for testing four groups and multiple ROIs (*p* = 0.05/(four groups × number of ROIs)).

## Results

### Group characteristics

No significant differences were present across groups with regard to age, handedness, education, BMI, ethnicity, or smoking (all *p* values > 0.1) (Table 1).

**Table 1:** Group characteristics

	Con-no-stress	Con-stress	Sib-no-stress	Sib-stress
*n*	19	20	20	19
Age (years)	32.6 (8.5)	34.8 (9.1)	33.8 (10.8)	32.5 (7.4)
Childhood maltreatment (CTQ total score)	34.4 (9.2)	35.7 (13.4)	36.2 (9.8)	34.0 (5.9)
Major life events (LSC-R total score)	4.3 (2.3)	4.6 (1.7)	4.8 (3.1)	4.9 (2.7)
Handedness (% right)	89.5	95	70	89.5
Education level	7.6 (2.7)	7.1 (1.9)	7.0 (1.6)	7.4 (1.5)
Body mass index	24.1 (2.7)	24.2 (2.1)	24.0 (3.0)	24.9 (3.9)
Ethnicity (% caucasian)	84.2	90	90	84.2
Smoker (% yes)	5.3	35	30	31.6

Mean values (SD) are denoted for age, education, and body mass index. All other values are reported in frequency

### Stress comparably increases cortisol, alpha-amylase levels, and subjective measures in controls and unaffected siblings

**Fig. 1:**
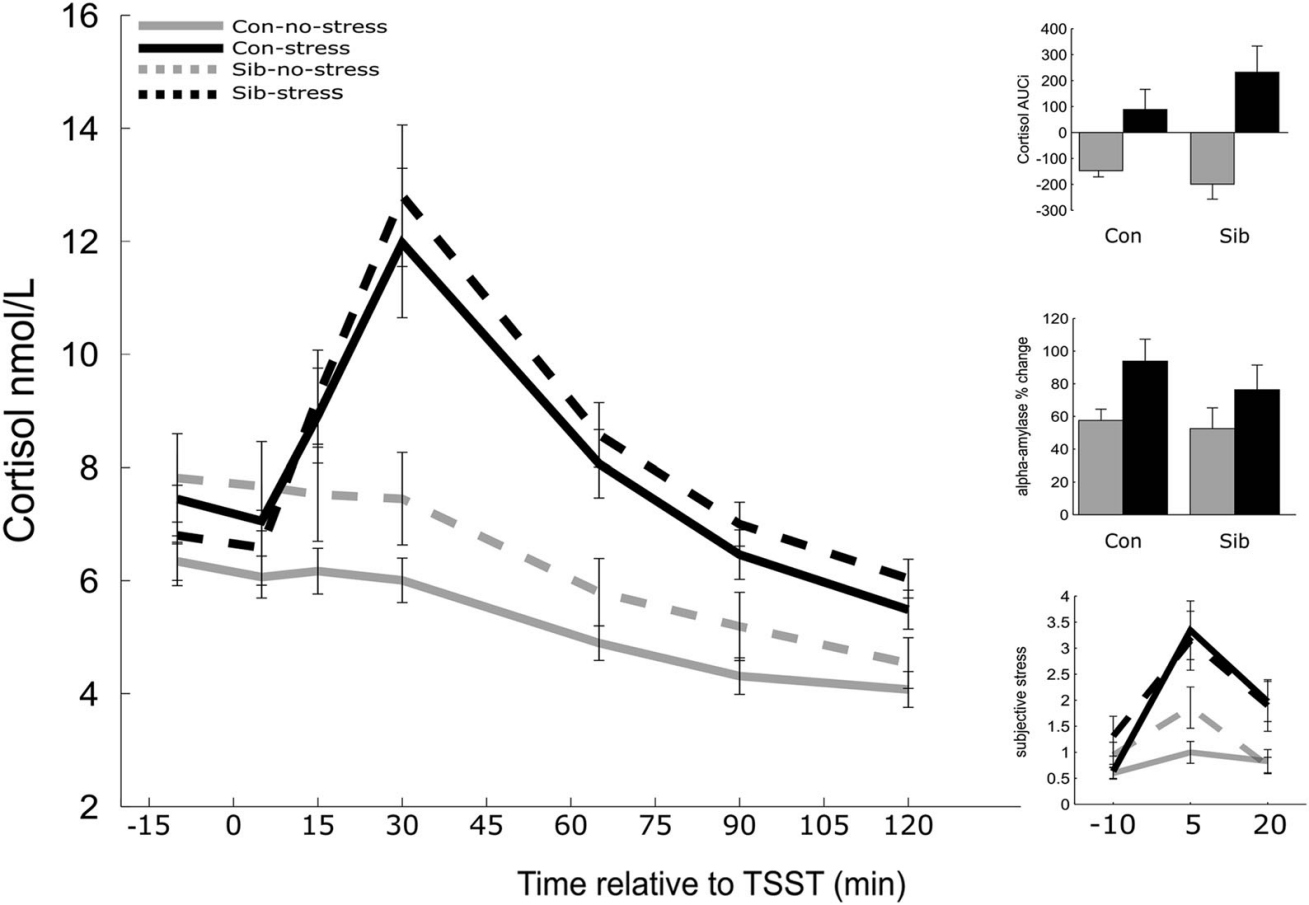
Endocrine, subjective, and autonomic stress measures. Con control, Sib schizophrenia sibling, TSST trier social stress test, AUCi area under the curve with respect to increase. Error bars represent standard error of the mean (SEM)

Stress-induced cortisol and alpha-amylase levels were comparable between controls and siblings (Fig. 1). Acute stress increased alpha-amylase (main effect of stress on percentage increase F(1,74) = 5.78, *p* = 0.019), cortisol (time × stress interaction, F(6,69) = 12.04, *p* < 0.001, main effect of stress on AUCi, F(1,74) = 22.251, *p* < 0.001), and subjective stress (time × stress interaction, F(2,72) = 9.43, *p* < 0.001). No significant differences were present between controls and siblings in stress-induced alpha-amylase, cortisol, or subjective stress (all *p* values > 0.05).

### Behavior

Stress did not significantly affect accuracy, reaction time, or head movement during the IAPS task, nor were there significant difference between controls and siblings in accuracy, reaction time, or head movement (Table S1) (all *p* values > 0.05).

### Responses to neutral, negative, and positive pictures following acute stress are different in at-risk individuals

**Fig. 2:**
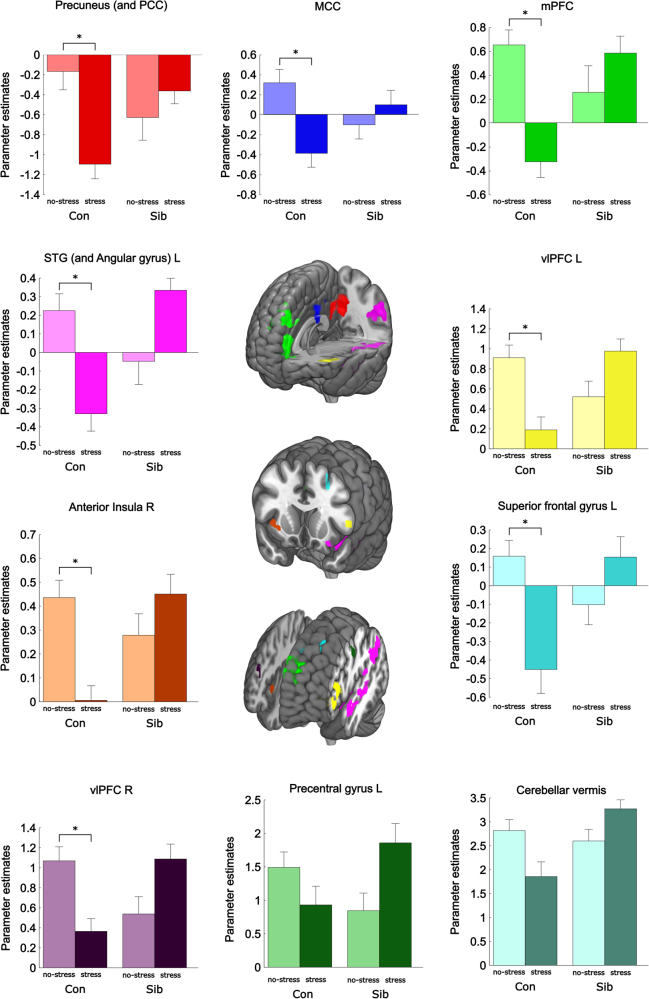
Significant clusters showing a group (control/sibling) × stress (stress/no-stress) interaction during the IAPS task after stress induction. Activation maps overlaid onto an anatomical scan in MNI-space (cluster-defining threshold of *p* < 0.001, cluster probability of *p* < 0.05, FWE-corrected). Con control, Sib schizophrenia sibling, PCC posterior cingulate cortex, MCC midcingulate cortex, mPFC medial prefrontal cortex, vlPFC ventrolateral prefrontal cortex, STG superior temporal gyrus, L left, R right. *X* and *z* coordinates refer to MNI coordinates. *survived Bonferroni correction of *p* < 0.00125 (*p* < 0.05/(four groups × ten ROIs)). Error bars represent standard error of the mean (SEM)

We performed whole-brain analyses to examine the differences in stress-induced brain responses between siblings and controls. We found no group × stress × valence interaction. We did find a group × stress interaction in the left superior frontal gyrus (SFG), left superior temporal gyrus (STG), precuneus/PCC, left angular gyrus, mPFC, bilateral ventrolateral prefrontal cortex (vlPFC), left precentral gyrus, cerebellar vermis, right anterior insula, and the midcingulate cortex (MCC) (Fig. 2 and Table 2). These results indicate that the effects of stress on subsequent responses to the pictures were not restricted to emotionally arousing stimuli. For our subsequent post hoc analyses, we therefore did not differentiate between valences.

**Table 2:** Brain areas showing an interaction between group (control/sibling) and stress (stress/no-stress) on brain responses to IAPS pictures after stress

Brain area	Side	Cluster size	Peak-voxel	MNI coordinates peak		
		mm^3^	*f* value	*x*	*y*	*z*
STG (extending to MTG and angular)	L	22518	57.37	−51	3	−12
Superior frontal	L	2673	55.14	−9	33	57
mPFC	L/R	17118	52.63	−12	57	33
Precuneus (extending to posterior cingulate)	L/R	5535	35.06	−12	−57	36
vlPFC	L	5724	31.87	−54	27	18
	R	4428	31.69	51	30	9
Precentral	L	1323	29.06	−51	−12	45
Cerebellar vermis	L/R	2727	26.21	9	−78	−27
Anterior insula	R	1512	23.62	36	12	−3
MCC	L/R	2808	22.55	0	−18	24

MNI coordinates represent the location of the peak voxels. Cluster-defining threshold of *p* < 0.001, cluster probability of *p* < 0.05, FWE-corrected

*STG* superior temporal gyrus, *MTG* middle temporal gyrus, *mPFC* medial prefrontal cortex, *vlPFC* ventrolateral prefrontal cortex, *MCC* midcingulate cortex, *L* left, *R* right, *MNI* Montreal Neurological Institute

Individual average brain activity for the three picture valences combined was extracted for each of these ROIs. Bonferroni-corrected post hoc comparisons between the four groups revealed a significant difference in eight regions between healthy controls in the no-stress and healthy controls in the stress condition, but not between siblings in the no-stress condition and siblings in the stress condition (Fig. 2 and Table S2). Moreover, activity within eight regions was significantly different between controls and siblings in the stress condition, but not in the no-stress condition. These results indicate that in these brain areas, the effects of stress on subsequent processing of environmental stimuli are different between healthy controls and siblings (Fig. 2 and Table S2). Valence-stratified analyses showed comparable results, indicating that the effect of stress was generalized to all stimuli and independent of valence (Figure S1).

To confirm that the results of the emotion processing task were consistent with previous literature [19, 32,], whole-brain analyses of negative versus neutral and positive versus neutral contrasts confirmed that the emotion task activated the expected emotion processing network in the control-no-stress group. Significant clusters were found in the occipital cortex, precuneus, middle temporal gyrus (MTG), ventromedial prefrontal cortex (vmPFC), amygdala, and hippocampus (Table S3).

## Discussion

This study investigated the effects of stress on subsequent brain responses in healthy controls and unaffected siblings of schizophrenia patients. We found that controls and siblings display large differences in brain activity in response to neutral and emotional pictures half an hour after acute stress, even though the endocrine, subjective, and autonomic stress responses were comparable. Following stress, core default mode network (DMN) regions and a region of the salience network (SN) were deactivated in healthy controls but not in schizophrenia siblings. We also identified regions outside of the DMN and SN that were suppressed after stress in controls but not in siblings, including the STG, MTG, MCC, vlPFC, precentral gyrus, and cerebellar vermis. To the best of our knowledge, this is the first study to show that stress-induced suppression of brain activity during the processing of pictures is extensively altered in siblings of schizophrenia patients who are at increased risk for a wide range of psychiatric disorders [11]. These results indicate that in healthy controls, other biological relevant processes compete for neuronal resources after stress [5], resulting in a suppression of self-referential processes, salience detection, but also emotional affect (vlPFC and cerebellar vermis) as well as motor functions (precentral gyrus) in response to neutral and emotional pictures.

The DMN is involved in self-referential processes such as mind-wandering, self-agency, and autobiographical memory retrieval [33]. Here, we found a significant difference between controls and siblings in brain responses following stress in core regions of the DMN, including the PCC, precuneus, angular gyrus, and mPFC, as well as the MCC, STG, and MTG. Although not included in the conventional DMN, the MCC, STG, and MTG are also activated during self-reference [34]. Activity within the DMN decreases during cognitively demanding tasks, promoting attention to external sensations rather than introspective processes [35]. Acute stress temporarily hampers this task-induced suppression of the DMN, increasing interference from internal emotional states, and thereby decreasing focused attention [6]. Later on, in the aftermath of stress, DMN connectivity decreases [9]. In the current study we found a robust deactivation of the DMN following stress in controls, but not in siblings. Several studies have demonstrated aberrant DMN activity in the absence of stress in several psychiatric disorders. First, both schizophrenia patients and relatives of patients failed to deactivate the DMN during rest as well as during a working memory task [36–40]. In addition, in schizophrenia patients, the normalization of DMN functional connectivity after antipsychotic treatment correlated with the change in illness severity [41] and poor DMN suppression is linked to feelings of hopelessness and rumination in remitted major depressive disorder patients [42, 43,]. Together, these results indicate that mental health is associated with the ability to deactivate the DMN and that an adaptive recovery from stress involves a dynamic shift away from the DMN after stress. In siblings, sustained activity within the DMN may result in increased rumination following stress and may be a precipitating factor in the development of psychopathology.

Our whole-brain analysis revealed that the right anterior insula deactivates following stress in controls, but not in unaffected siblings of schizophrenia patients. The anterior insula is part of the SN [44]. The SN is involved in the detection of salient stimuli and the rapid generation of behavioral responses to these stimuli by switching between functional networks [44, 45,]. An adaptive stress response involves the reallocation of neuronal resources to the SN during stress, improving threat detection and promoting survival by taking rapid actions [5], and an adequate termination of these responses in the aftermath of stress, promoting adaptation [46]. Sustained activation of this area after stress might lead to a chronic state of hypervigilance and predispose an individual to develop psychopathology on the longer term. However, we cannot exclude the possibility that in schizophrenia siblings, this is an adaptive, compensatory neuronal mechanism, which may have prevented the development of psychopathology.

In the present study, stress-induced neuronal deactivation was independent of the valence of the stimulus, and comparable results were found across neutral, negative, and positive pictures. These results are in line with previous studies that found reduced responsiveness of the amygdala [8] and reduced acoustic startle reflex [47] after exogenous cortisol administration, both independent on valence. These findings indicate that corticosteroids aid an adequate termination or limitation of the stress response, protecting the organism against the detrimental effects of stress. We suggest that in healthy controls, cortisol nonspecifically attenuates DMN and SN responses to emotional and neutral pictures and thereby reduces vigilance and interference of internal emotional states. It should be taken into account though that the brain regions that are differentially affected by stress between groups are not necessarily task-specific regions and therefore it should be considered that other tasks might be more specific to the observed effect.

Another possible explanation for the neuronal suppression after stress in healthy controls is mental exhaustion or distraction. However, we consider this possibility unlikely since Esposito et al. [48] found that self-reported exhaustion ratings after prolonged mental performance were associated with increased DMN connectivity. Moreover, reaction time and accuracy were comparable across groups.

Although it has been suggested that HPA-axis activity is related to the genetic risk for schizophrenia [49], we found that the stress-induced cortisol response was not different between healthy controls and schizophrenia siblings. Despite comparable endocrine and behavioral outcomes, brain activity at the peak of stress-induced cortisol levels was significantly different, highlighting the importance of performing multimodal research in order to understand the susceptibility to stress-related psychopathology.

Our study has several strengths. We carefully selected a group of unaffected siblings and matched healthy controls with comparable trauma scores. We excluded any current psychiatric disorders as well as medication that could have influenced the cortisol response. However, there are also limitations. First, we only included male subjects which weakens the generalizability of the results. Second, 34 out of 39 siblings were recruited through advertisements. In these, the diagnosis of schizophrenia could not officially be confirmed due to privacy regulations. However, since we extensively asked siblings about characteristics of the disorder in their affected sibling, the likelihood of false positives is very small. Third, although parameter estimates for all valences were analyzed separately, and thereby the misinterpretation of our results caused by differential responses to one of the valences was avoided, it cannot be ruled out that reduced parameters estimates in the control-stress group were caused by increased activity during rest (fixation cross). Fourth, we did not assess subclinical symptoms in the participants. Although psychiatric disorders were excluded in all participants, subclinical symptoms may have been higher in the unaffected siblings as compared to the healthy controls. However, a previous study in a Dutch sibling sample did not find any differences in the positive, negative, and depressive dimensions of the community assessment of psychic experiences (CAPE) [50]. Therefore, we do not expect that large or clinically meaningful differences in symptoms may have been present in our sample. Finally, we only included one specific at-risk group, and it is unknown whether the results in these individuals can be extrapolated to other at-risk groups. Future longitudinal studies are warranted to examine whether differential patterns of brain activity following a stress manipulation predict negative outcomes after chronic or acute stress.

In conclusion, our results show that in healthy controls, acute stress results in the suppression of several brain areas including the DMN half an hour later, suggesting that an adequate response to acute stress involves a dynamic and widespread reallocation of neuronal resources after stress. However, this neuronal reallocation was absent in unaffected siblings of schizophrenia patients, indicating that sustained responses of these brain areas following stress may increase the vulnerability to stress in these at-risk individuals.

## Electronic supplementary material

Table S1

Table S2

Table S3

Figure S1

## Electronic supplementary material

**Supplementary information** accompanies this paper at 10.1038/s41386-018-0026-8 contains supplementary material.
