# Supplementary material for: At-risk individuals display altered brain activity following stress
Source: Neuropsychopharmacology. 2018 Feb 26;43(9):1954–60. doi: 10.1038/s41386-018-0026-8 (PMC6046038; doi:10.1038/s41386-018-0026-8)
Supplement: Supplementary file 1 — Table S1 [file 41386_2018_26_MOESM1_ESM.docx]

|  |  | Con-no-stress | Con-stress | Sib-no-stress | Sib-stress |
| --- | --- | --- | --- | --- | --- |
| Accuracy in % ± SEM | *Neutral* | 71 ± 4.4% | 65 ± 2.9% | 71 ± 3.7% | 65 ± 4.3% |
|  | *Negative* | 92 ± 1.9% | 90 ± 2.9% | 93 ± 1.8% | 91 ± 1. 8% |
|  | *Positive* | 84 ± 3.4% | 83 ± 2.5% | 86 ± 2.4% | 85 ± 2.6% |
| Reaction times in ms ± SEM | *Neutral* | 656 ± 46 ms | 637 ±51 ms | 658 ± 42 ms | 727 ± 55 ms |
|  | *Negative* | 562 ± 35 ms | 559 ± 43 ms | 544 ± 28 ms | 641 ± 54 ms |
|  | *Positive* | 628 ± 38 ms | 562 ± 44 ms | 592 ± 39 ms | 692 ± 48 ms |
| Max head movement in mm ± SEM |  | 0.28 ± 0.20 | 0.44 ± 0.37 | 0.37 ± 0.31 | 0.41 ± 0.29 |

**Table S1 | Performance and head movement during emotion processing task.** Accuracy: percentage of trials that agreed with IAPS ratings. Reactions times represent reaction times for trials that agreed with IAPS ratings. Max head movement represents the mean of the maximum scan-to-scan head movement.
