# Supplementary material for: At-risk individuals display altered brain activity following stress
Source: Neuropsychopharmacology. 2018 Feb 26;43(9):1954–60. doi: 10.1038/s41386-018-0026-8 (PMC6046038; doi:10.1038/s41386-018-0026-8)
Supplement: Supplementary file 2 — Table S2 [file 41386_2018_26_MOESM2_ESM.docx]

| Brain area | Con-no-stress vs  Con-stress | | Con-no-stress vs  Sib-no-stress | | Sib-no-stress vs  Sib-stress | | Con-stress vs  Sib-stress | |
| --- | --- | --- | --- | --- | --- | --- | --- | --- |
|  | T | p | T | P | T | P | T | P |
| STG L | 4.26 | < 0.001* | 1.76 | 0.087 | -2.69 | 0.011 | -5.79 | < 0.001* |
| Superior frontal L | 3.90 | < 0.001* | 1.90 | 0.066 | -1.67 | 0.103 | -3.56 | 0.001* |
| mPFC | 5.37 | < 0.001* | 1.54 | 0.133 | -1.23 | 0.225 | -4.70 | < 0.001* |
| Precuneus | 3.99 | < 0.001* | 1.57 | 0.125 | -1.00 | 0.322 | -3.76 | 0.001* |
| vlPFC L | 3.97 | < 0.001* | 1.93 | 0.062 | -2.29 | 0.028 | -4.43 | < 0.001* |
| vlPFC R | 4.58 | 0.001* | 2.37 | 0.023 | -2.40 | 0.022 | -3.70 | 0.001* |
| Precentral L | 1.54 | 0.133 | 1.84 | 0.074 | -2.60 | 0.013 | -2.31 | 0.027 |
| Cerebellar vermis | 2.49 | 0.017 | 0.65 | 0.520 | -2.17 | 0.036 | -3.88 | < 0.001* |
| Anterior insula R | 4.56 | < 0.001* | 1.36 | 0.183 | -1.41 | 0.167 | -4.36 | < 0.001* |
| MCC | 3.65 | 0.001* | 2.15 | 0.039 | -0.99 | 0.331 | -2.42 | 0.020 |

**Table S2 | Two-samples T-tests on regions that showed a group * stress interaction.** STG: superior temporal gyrus; mPFC: medial prefrontal cortex; vlPFC: ventrolateral prefrontal cortex; MCC: midcingulate cortex; L: Left; R: Right. * survived Bonferroni correction of p<0.00125 (p<0.05/(four groups * ten ROIs)).
