# Supplementary material for: At-risk individuals display altered brain activity following stress
Source: Neuropsychopharmacology. 2018 Feb 26;43(9):1954–60. doi: 10.1038/s41386-018-0026-8 (PMC6046038; doi:10.1038/s41386-018-0026-8)
Supplement: Supplementary file 3 — Table S3 [file 41386_2018_26_MOESM3_ESM.docx]

| Brain area | Side | Cluster size  mm^3^ | T-value | MNI coordinates peak *x y z* | | |
| --- | --- | --- | --- | --- | --- | --- |
| Negative vs. neutral |  |  |  |  |  |  |
| Inferior occipital (extending to MTG) | R | 9621 | 12.87 | 54 | -69 | 3 |
| Middle occipital (extending to MTG and fusiform) | L | 4599 | 11.17 | -48 | -69 | 12 |
| Precuneus | L/R | 3384 | 8.22 | 3 | -54 | 24 |
| Amygdala (extending to hippocampus) | R | 504 | 5.95 | 21 | -9 | -12 |
| MTG | R | 594 | 5.76 | 51 | -9 | -18 |
| Occipital pole (extending to superior occipital) | R | 828 | 5.59 | 12 | -93 | 9 |
| Calcarine (extending to cuneus) | L | 549 | 5.31 | -12 | -84 | -3 |
| Positive vs. neutral |  |  |  |  |  |  |
| Occipital fusiform | L/R | 12420 | 9.48 | -27 | -66 | -9 |
| Anterior cingulate (extending to vmPFC) | L/R | 1431 | 7.51 | 0 | 42 | -6 |
| Inferior occipital (extending to MTG) | L | 1206 | 7.28 | -45 | -72 | 6 |
| Hippocampus (extending to amygdala) | R | 306 | 7.03 | 30 | -9 | -18 |
| Precuneus (extending to lingual) | R | 1251 | 4.63 | 9 | -57 | 15 |

**Table S3 | Significant clusters during the viewing of negative versus neutral and positive versus neutral pictures.** MNI coordinates represent the location of the peak voxels. Cluster-defining threshold of p<0.001, cluster-probability of p<0.05, FWE-corrected. L: Left; R: Right; MNI: Montreal Neurological Institute; MTG: Middle temporal gyrus; vmPFC: ventromedial prefrontal cortex.
