# Supplementary material for: At-risk individuals display altered brain activity following stress
Source: Neuropsychopharmacology. 2018 Feb 26;43(9):1954–60. doi: 10.1038/s41386-018-0026-8 (PMC6046038; doi:10.1038/s41386-018-0026-8)
Supplement: Supplementary file 4 — Figure S1 [file 41386_2018_26_MOESM4_ESM.docx]

**
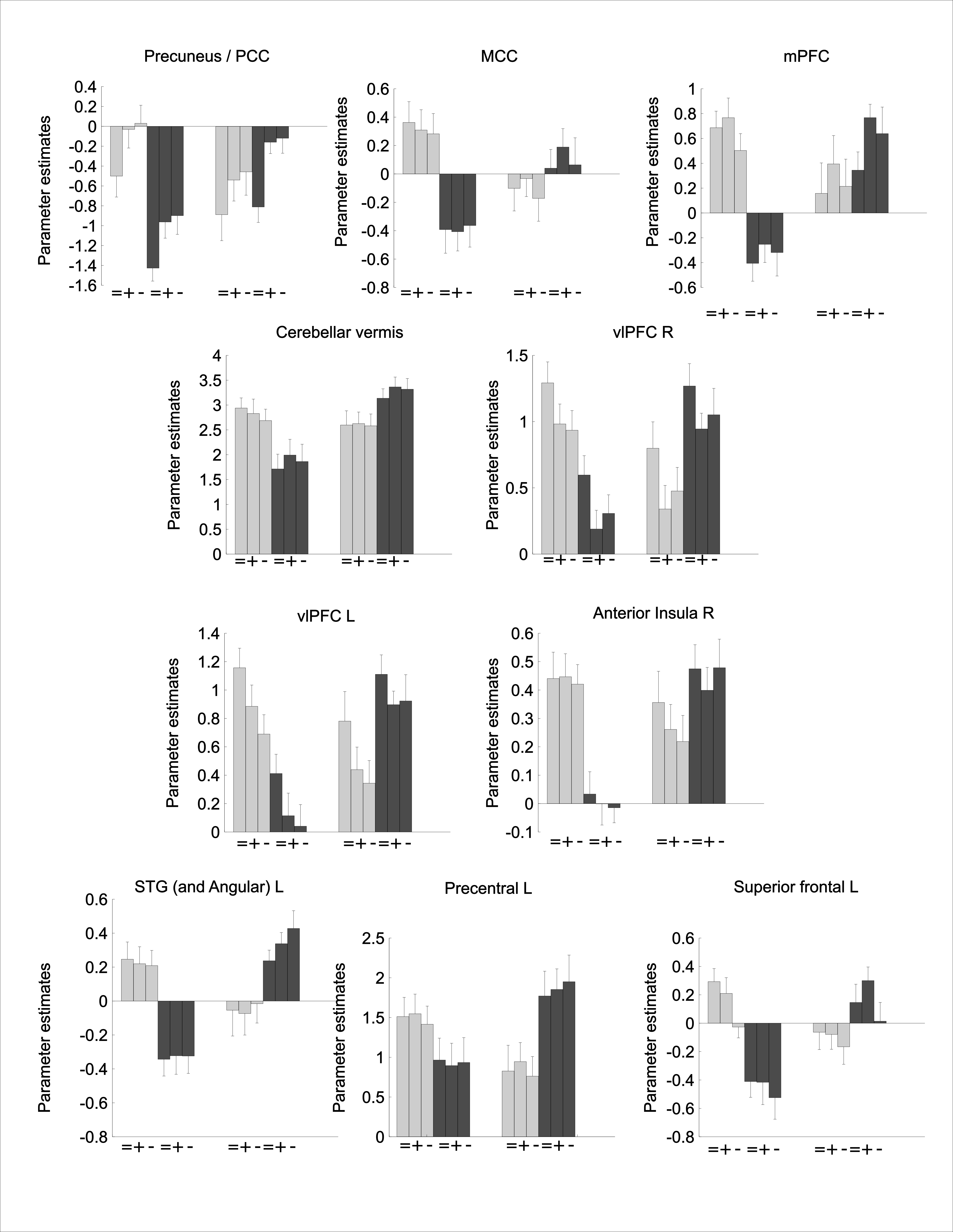
**

**Figure S1 | Clusters showing a group (control/sibling) * stress (stress/no-stress) interaction, each valence presented separately.** Con:  Control; Sib: Schizophrenia sibling; PCC: posterior cingulate cortex; mPFC: medial prefrontal cortex; STG: superior temporal gyrus; vlPFC: ventrolateral prefrontal cortex; =: neutral; +: positive; -: negative. Error bars represent standard error of the mean (SEM).
